# Socioeconomic Inequalities in Dietary and Physical Activity Behaviours Among Adolescents: A Systematic Review

**DOI:** 10.1111/jhn.70374

**Published:** 2026-11-02

**Authors:** Mariana R. Haddad, Flávia M. Sarti

**Affiliations:** ^1^ Department of Nutrition (DN) Federal University of Espírito Santo (UFES) Vitória Espírito Santo Brazil; ^2^ School of Arts, Sciences and Humanities (EACH) University of São Paulo (USP) São Paulo São Paulo Brazil

**Keywords:** adolescent, exercise, feeding behaviour, health inequities, social determinants of health, socioeconomic factors

## Abstract

**Objectives:**

To synthesize the available evidence on the association between socioeconomic position and adolescents' dietary behaviours and physical activity.

**Study Design:**

Systematic review.

**Methods:**

This systematic review was conducted in accordance with the PRISMA 2020 guidelines. PubMed/MEDLINE, Scopus, Embase, and BIREME were searched for observational studies published between January 2005 and February 2026 that examined the association between indicators of socioeconomic position and adolescents' dietary behaviours or physical activity. Methodological quality was assessed using the Joanna Briggs Institute critical appraisal checklists.

**Results:**

A total of 22,897 records were identified, of which 38 studies met the eligibility criteria and were included in the review. The findings showed socioeconomic inequalities in adolescents' lifestyle behaviours. Higher socioeconomic position was associated with healthier dietary patterns, greater fruit and vegetable intake, and greater participation in organized physical activities. In contrast, lower socioeconomic position was associated with poorer diet quality, more frequent breakfast skipping, and higher consumption of energy‐dense or ultra‐processed foods, whereas associations with total physical activity and sedentary behaviour were inconsistent. Parental education showed the most consistent associations with dietary behaviours. Methodological quality was rated as high in 15 studies, moderate in 13, and low in 10.

**Conclusions:**

Socioeconomic position is consistently associated with adolescents' dietary behaviours, whereas its associations with physical activity and sedentary behaviour vary by domain and setting. Reducing these inequalities will require public health strategies that improve equitable access to healthy food environments and safe opportunities for physical activity while addressing the broader social and environmental determinants of health.

## Practitioner Points

- Socioeconomic position is consistently associated with adolescents' dietary behaviours across diverse settings, whereas associations with physical activity and sedentary behaviour are less consistent; most of the evidence is cross‐sectional.
- Parental education is the socioeconomic indicator most consistently associated with adolescents' dietary behaviours; findings for household resources and neighbourhood‐level indicators are more heterogeneous.
- Public health strategies should address structural social determinants to reduce health inequalities and promote healthier developmental trajectories.

## 1 Introduction

Adolescence is a critical developmental stage marked by profound biological, psychological, and social changes [1]. During this period, key health behaviours, including dietary habits and physical activity, are established and frequently persist into adulthood [2]. Consequently, adolescence represents a pivotal window for health promotion and disease prevention [3]. Health behaviours adopted during these formative years influence well‐being across the life course and contribute to the development of chronic non‐communicable diseases (NCDs) later in life [4, 5, 6]. Indeed, dietary risks remain among the leading contributors to the global burden of disease, and low physical activity also contributes to this burden [7].

Despite growing recognition of the importance of healthy lifestyles during adolescence, poor dietary habits and insufficient physical activity remain highly prevalent worldwide. International surveillance data consistently indicate that a substantial proportion of adolescents do not meet recommended levels of physical activity (81% of school‐going adolescents aged 11–17 years worldwide in 2016), contributing to increasing rates of overweight, obesity, and metabolic disorders [4, 7, 8, 9]. Concurrently, changes in food systems, rapid urbanization, and the widespread availability of ultra‐processed foods have substantially transformed dietary patterns, promoting greater consumption of energy‐dense, nutrient‐poor food products [10, 11, 12].

Dietary behaviours and physical activity are closely interconnected and play a fundamental role in adolescent health and well‐being [13]. However, opportunities to adopt healthy lifestyle behaviours are unequally distributed and are strongly shaped by socioeconomic position (SEP) [14, 15]. Adolescents from lower‐SEP households often face multiple barriers to healthy living, including limited access to nutritious foods, unsafe environments for outdoor activities, and fewer opportunities to participate in organized sports and other recreational activities. They are also more likely to engage in prolonged sedentary behaviours [16, 17]. In contrast, adolescents from higher‐SEP households generally have better access to healthy food environments, recreational facilities, and resources that facilitate healthier lifestyle choices [14, 16]. Nevertheless, lower SEP operates as a population‐level risk factor rather than a deterministic attribute of individual adolescents: not all adolescents from lower‐SEP households present less healthy behaviours [18], and the focus of this review is on the social inequalities that structure opportunities for healthy living rather than on the characteristics of particular groups of adolescents.

From a life‐course perspective, socioeconomic inequalities experienced during adolescence may have enduring consequences, contributing to health disparities that extend into adulthood and may even be transmitted across generations [19, 20, 21]. These inequalities are influenced not only by household‐level resources but also by broader structural determinants, including educational opportunities, income distribution, and social policies [21, 22].

Research examining the association between SEP and adolescents' dietary and physical activity behaviours has grown considerably over the past two decades. Previous systematic reviews have synthesized evidence on these associations [14, 15]. However, a substantial body of new evidence has since emerged from countries with diverse socioeconomic contexts. Furthermore, SEP has been assessed using a variety of indicators, including parental education, household income, family affluence, and neighbourhood characteristics. An updated synthesis of this evidence is therefore warranted to determine whether these associations are consistent across different populations and settings, to identify gaps in the current literature, and to inform future research and public health strategies.

SEP refers to the social and economic factors that influence the positions individuals or groups hold within the structure of society [23]. Because adolescents rarely have their own income, education, or occupation, their SEP is usually assigned through household or area characteristics, and each indicator captures a partly distinct dimension with plausibly different pathways to diet and physical activity [23, 24]. Parental education, usually reported by parents or adolescents, reflects knowledge‐related resources such as health and nutrition literacy, which may shape household food choices, meal routines, and attitudes toward active living. Household income and wealth, usually reported by parents, reflect the material resources that determine the affordability of nutritious foods, sports club fees, and equipment. Parental occupation captures social standing, working conditions, and the time available for meal preparation and for supporting structured activities. The Family Affluence Scale (FAS), now in its third version (FAS III), is reported by adolescents themselves and summarizes household material assets (e.g., car and computer ownership, having one's own bedroom, and family holidays), offering a practical alternative when adolescents cannot accurately report parental income or education [25, 26]. Composite indices combine two or more of these dimensions into a single score or classification (e.g., national socioeconomic strata based on household assets and the education of the head of household), and derived measures such as the family income‐to‐poverty ratio adjust income for household size and poverty thresholds. Finally, area‐level indicators, such as neighbourhood deprivation indices or median neighbourhood income, capture the food, built, and safety environments in which adolescents live and may influence behaviours independently of household resources [24].

Physical activity and sedentary behaviour are related but distinct constructs. Physical activity is defined as any bodily movement produced by skeletal muscles that results in energy expenditure [27], whereas sedentary behaviour is any waking behaviour characterized by low energy expenditure (≤ 1.5 metabolic equivalents) while sitting, reclining, or lying, such as recreational screen time [28]. Physical inactivity, in turn, refers to not meeting current physical activity recommendations [28]. An adolescent can therefore meet physical activity recommendations and still accumulate long periods of sedentary time. Both behaviours matter for long‐term health through partly different routes: physical activity supports cardiorespiratory and muscular fitness, bone health, and cardiometabolic health, whereas higher amounts of sedentary behaviour, particularly recreational screen time, are associated with greater adiposity, poorer cardiometabolic health, and shorter sleep duration [9]. Dietary behaviours, in turn, range from the consumption of specific foods or food groups (e.g., fruit and vegetables or ultra‐processed foods) and meal patterns (e.g., breakfast skipping) to overall diet quality, a multidimensional construct reflecting the adequacy, variety, moderation, and balance of the diet relative to dietary recommendations [29].

Accordingly, this systematic review aimed to synthesize the available evidence on the association between SEP and adolescents' dietary behaviours and physical activity patterns across diverse countries and socioeconomic contexts. By synthesizing studies conducted across diverse geographic and socioeconomic settings and considering multiple indicators of SEP, this review provides an updated overview of the associations between socioeconomic inequalities and lifestyle behaviours during adolescence. A clearer understanding of these associations may inform public health policies and interventions designed to reduce health inequalities, promote healthier lifestyles, and improve health outcomes throughout the life course.

## 2 Methods

### 2.1 Study Design

This systematic review was conducted in accordance with the Preferred Reporting Items for Systematic Reviews and Meta‐Analyses (PRISMA 2020) guidelines [30]. The search strategy was also reported following the PRISMA‐S recommendations [31]. The review protocol was prospectively registered in the International Prospective Register of Systematic Reviews (PROSPERO; registration number CRD42016036162). Although the protocol was registered in 2016, the literature search was updated immediately before the review was conducted to ensure that the synthesis reflected the most recent available evidence.

### 2.2 Search Strategy

A comprehensive literature search was conducted in PubMed/MEDLINE, Scopus, Embase, and BIREME. The search strategy combined controlled vocabulary (Medical Subject Headings [MeSH]) and free‐text terms related to adolescents, SEP, dietary behaviours, and physical activity. Boolean operators (AND and OR) were used to combine the search terms. Because ‘socioeconomic status’ (SES) remains the most frequently used term in this literature, SEP was searched using both ‘socioeconomic status’ and ‘socioeconomic position’ (the latter in PubMed, Scopus, and Embase), together with indicator‐specific terms (Additional File S1); throughout this review, the term SEP is used to refer to all of these measures.

Studies published from January 2005 to February 2026 were eligible for inclusion. The final search was performed on 15 February 2026. Search strategies were adapted for each database to account for differences in indexing systems and search syntax. The complete search strategies are provided in Additional File S1. To maximize the retrieval of relevant studies, the reference lists of all included articles were also screened manually.

### 2.3 Eligibility Criteria

Eligibility criteria were defined using the PICOS framework (Population, Exposure, Comparison, Outcomes, and Study design) [32], as summarized in Box 1.

Box 1 **PICOS framework used to define the research question and eligibility criteria of the systematic review.**

**Table:** 

PICOS element	Description
Population	Adolescents aged 10–19 years, of either sex, from studies conducted in any country.
Exposure	Indicators of socioeconomic position (SEP): parental education, household income or wealth, parental occupation, family affluence/assets, composite socioeconomic indices, and area‐level (neighbourhood) socioeconomic indicators.
Comparison	Adolescents from different socioeconomic groups or categories (e.g., low, middle, and high SEP).
Outcomes	Dietary behaviours (including dietary patterns or food consumption), physical activity, and sedentary behaviour.
Study design	Observational studies, including cross‐sectional and cohort designs, based on primary or secondary data analyses examining associations between socioeconomic indicators and adolescents’ dietary behaviours and/or physical activity.

Abbreviations: PICOS, Population, Exposure, Comparison, Outcomes, and Study design; SEP, socioeconomic position.

*Source:* Prepared by the authors.

The population of interest comprised adolescents aged 10–19 years, according to the World Health Organization (WHO) definition of adolescence [56]. Studies including broader age ranges were eligible when data for adolescents could be extracted separately.

The exposures of interest were conventional indicators of SEP measured at the household or area level [23, 24]: (i) parental education (e.g., highest level or years of schooling completed by the mother, father, or either parent); (ii) household income or wealth (e.g., absolute or equivalized income, income quintiles, income‐to‐poverty ratio, or asset‐based wealth indices); (iii) parental occupation or employment status (e.g., occupational social class); (iv) family affluence, assessed mainly with the adolescent‐reported FAS; (v) composite socioeconomic indices combining two or more of these dimensions (e.g., national socioeconomic classifications or SES scores derived from education, occupation, and income); and (vi) area‐level socioeconomic indicators (e.g., neighbourhood deprivation indices or median neighbourhood income). Other sociodemographic variables reported by some studies, such as urban or rural residence, school type, ethnicity, family structure, geographic region, or nutrition knowledge, were not considered SEP indicators; in the synthesis, only associations involving the conventional indicators listed above were considered. Three studies were conducted exclusively among adolescents from lower‐SEP households, with SEP used as a sampling criterion rather than as a comparison between groups [34, 39, 40]. These studies were retained because they provide evidence on factors associated with healthier behaviours within lower‐SEP groups (e.g., dietary resilience); however, because they did not compare adolescents across socioeconomic groups, they were not used to infer socioeconomic gradients and are discussed separately. The outcomes of interest included dietary behaviours and physical activity patterns. Dietary outcomes comprised dietary patterns, food consumption, fruit and vegetable intake, breakfast consumption, and overall diet quality. Diet quality was defined as the overall healthfulness of the diet relative to dietary recommendations, assessed with a priori indices (e.g., Healthy Eating Index, KIDMED, or dietary diversity scores) or with a posteriori dietary patterns derived from food frequency questionnaires or 24‐h recalls. Physical activity outcomes included overall physical activity levels, participation in organized sports, leisure‐time physical activity, and sedentary behaviours. Physical activity was defined as any bodily movement produced by skeletal muscles that results in energy expenditure [27], including total or moderate‐to‐vigorous physical activity, organized sports participation, and leisure‐time activity, assessed by questionnaire, diary, or accelerometry. Sedentary behaviour was defined as waking behaviour with low energy expenditure (≤ 1.5 metabolic equivalents) in a sitting, reclining, or lying posture [28], most often operationalized in the included studies as screen time (television, computer, or video games).

Observational studies with cross‐sectional or cohort designs were eligible for inclusion. Studies published in English or Portuguese and available in full text were included if they reported empirical data on the association between SEP and adolescents' dietary behaviours or physical activity patterns. Review articles, editorials, conference abstracts, studies conducted exclusively in clinical populations, and publications without primary data were excluded.

### 2.4 Study Selection and Data Extraction

Records identified through the database searches were imported into Zotero for reference management and duplicate removal. The remaining records were then exported to Rayyan, an online platform designed for study screening in systematic reviews [57]. Before screening, reviewers completed a calibration exercise using a sample of studies to ensure consistent application of the eligibility criteria [58].

Two reviewers independently screened titles and abstracts to identify potentially eligible studies. Full‐text articles were subsequently assessed against the predefined inclusion and exclusion criteria. Disagreements were resolved through discussion, and a third reviewer was consulted whenever consensus could not be reached.

Data extraction was performed independently by two reviewers using a standardized and pilot‐tested extraction form. The following data were extracted from each study: authors and year of publication, country, study design, sample characteristics, indicators of SEP, methods used to assess dietary behaviours or physical activity, and the main findings. The study selection process is presented in the PRISMA 2020 flow diagram (Figure 1).

**Figure 1:**
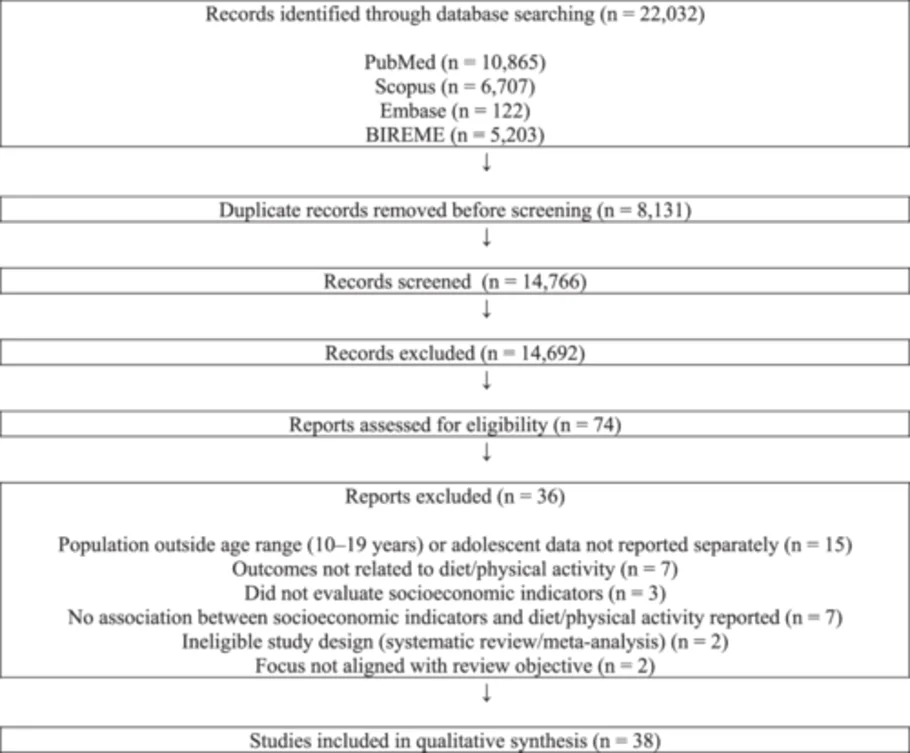
PRISMA 2020 flow diagram of the study selection process.

### 2.5 Quality Assessment and Data Synthesis

Methodological quality was assessed using the Joanna Briggs Institute (JBI) Critical Appraisal Checklists for Analytical Cross‐Sectional and Cohort Studies [58]. Two reviewers independently appraised each study, and any disagreements were resolved through discussion until consensus was reached.

Because the checklists differ in the number of items (8 for analytical cross‐sectional studies and 11 for cohort studies), each study was scored as the proportion of applicable criteria met and classified as high (≥ 70%), moderate (50%–69%), or low (< 50%) methodological quality. Items rated as unclear were counted as not met. Items that were not applicable were excluded from the denominator: the item on objective, standard criteria was considered not applicable when outcomes were analyzed as continuous variables, and the items on comparability of exposure groups were considered not applicable in cohorts restricted to a single socioeconomic stratum. Item‐level ratings are provided in Additional File S2.

Given the substantial heterogeneity in socioeconomic indicators, behavioural outcomes, and methodological characteristics across the included studies, quantitative synthesis was considered inappropriate [58]. Therefore, the findings were synthesized narratively, with emphasis on study characteristics and the reported associations between SEP and adolescents' dietary behaviours and physical activity patterns. Within each outcome domain, the synthesis was organized by type of SEP indicator.

## 3 Results

### 3.1 Study Selection

The study selection process is presented in Figure 1. A total of 22,897 records were retrieved from PubMed (*n* = 10,865), Scopus (*n* = 6707), Embase (*n* = 122), and BIREME (*n* = 5203). The complete search strategies for each database are presented in Additional File S1.

Following duplicate removal, 14,766 records remained for title and abstract screening. Most records were excluded during the initial screening, leaving 74 articles for full‐text assessment. Of these, 36 studies were excluded because they included populations outside the predefined age range, did not assess dietary behaviours or physical activity outcomes, did not evaluate indicators of SEP, did not meet the predefined study design criteria, or did not report associations between SEP and dietary or physical activity outcomes.

A total of 38 studies met the eligibility criteria and were included in the qualitative synthesis.

### 3.2 Characteristics of Included Studies

The 38 included studies were published between 2005 and 2025 and represented diverse geographic settings: Europe (*n* = 15), North America (*n* = 4), Oceania (*n* = 6, all from Australia), South America (*n* = 4), Asia (*n* = 4), and Africa (*n* = 3), with two additional multi‐country studies spanning more than one region [18, 33, 34, 35, 36, 37, 38, 39, 40, 41, 42, 43, 44, 45, 46, 47, 48, 49, 50, 51, 52, 53, 54, 55, 59, 60, 61, 62, 63, 64, 65, 66, 67, 68, 69, 70, 71, 72]. Most studies employed a cross‐sectional design (*n* = 31), two used repeated cross‐sectional or trend analyses, and five were longitudinal. Among the latter, follow‐up periods ranged from 1 to 2 years in four studies, whereas one birth cohort followed participants from age 5 to 17 years. Sample sizes ranged from 127 participants in small school‐based investigations to 109,104 adolescents in nationally representative surveys.

Participants were generally between 10 and 19 years of age, and most studies included both boys and girls recruited through school settings. SEP was assessed using a variety of indicators (Table 1). Parental education was the most frequently used indicator (*n* = 20 studies), followed by parental occupation or employment status (*n* = 10), family affluence or household material resources (*n* = 10), household income or wealth (*n* = 10), composite or global SES measures, including perceived SEP (*n* = 5), and area‐ or school‐level socioeconomic indicators (*n* = 5). Eighteen studies (47%) examined two or more SEP indicators, and another 17 relied on a single indicator. Three studies were conducted exclusively among adolescents from lower‐SEP households and therefore examined correlates of behaviour within, rather than across, socioeconomic groups [34, 39, 40].

**Table 1:** Main characteristics and findings of studies included in the systematic review on socioeconomic factors associated with diet, physical activity, and health‐related behaviours among adolescents.

Study	Design and sample	SEP indicators	Instruments and outcomes (Diet/PA)	Main results	Quality assessment (JBI)
Frenn et al. [33], United States	CS; *n* = 127; African American and Hispanic adolescents (~12–13 years).	Family income (free‐lunch eligibility); ZIP code of residence (not an SEP indicator).	Diet: percentage of energy from fat (Food Habits Questionnaire). PA: 3‐day activity log (CAAL); psychosocial antecedents (Health Promotion/Transtheoretical Model).	Family income was not associated with PA or dietary fat, but family role models and support for PA and a low‐fat diet increased with family income (*p* < 0.05). Total social support was associated with higher PA among girls.	3/7 (low quality). Not met or unclear: inclusion criteria (1), description of participants and setting (2), SEP measurement (3), statistical analysis (8). Item 4 not applicable (continuous outcome).
Li and Wang [34], United States (Baltimore)	Long.; 1‐year follow‐up; *n* = 181; 10–14 years; 57% girls.	None analyzed (sample restricted to low‐income African American households).	Diet: 152‐item FFQ assessing dietary patterns. PA: self‐reported PA.	The Western dietary pattern showed moderate tracking over time (*r* = 0.47; *p* < 0.001), indicating persistence of unhealthy eating behaviours. Age, sex, PA, and BMI predicted dietary changes; no comparison across socioeconomic groups.	4/9 (low quality). Not met or unclear: SEP measurement (3), outcome measurement (7), completeness of follow‐up (9), strategies for incomplete follow‐up (10), statistical analysis (11). Items 1–2 not applicable (single socioeconomic stratum).
Aounallah‐Skhiri et al. [35], Tunisia	CS; clustered random sample from three regions (2005); *n* = 1019; 15–19 years.	Household economic level (asset‐based index), parental education and profession, and mother working outside the home; urban/rural residence.	Diet: 134‐item semi‐quantitative FFQ; DQI‐I; dietary patterns derived by multiple correspondence analysis. PA assessed as a covariate.	After mutual adjustment, a ‘modern’ dietary pattern (more white bread, sugars, added fats, and dairy; fewer oils, grains, legumes, and vegetables) increased with household economic level (upper vs. lower: +14.9; 95% CI, 10.8–19.0) and with higher maternal education (*p* = 0.0013). The modern pattern was associated with overweight in boys (3rd vs. 1st tertile: POR = 3.3; 95% CI, 1.3–8.7).	6/7 (high quality). Not met or unclear: SEP measurement (3). Item 4 not applicable (continuous outcome).
Stephens et al. [18], Australia	CS; *n* = 2171 (1014 with low maternal education; 55% girls); 12–15 years.	Maternal education (low, medium, high); multivariable analyses restricted to adolescents with low maternal education.	Diet: fruit (≥ 1 time/day) and vegetable consumption (≥ 2 times/day) assessed using an FFQ.	Frequent fruit intake increased with maternal education (low, medium, high: boys 27%, 30%, 49%; girls 36%, 42%, 54%; *p* < 0.001), as did frequent vegetable intake among girls (29%, 30%, 38%; *p* = 0.021) but not boys. Among low‐SEP adolescents, greater fruit and vegetable intake was associated with perceived importance of health and being served vegetables at dinner (*p* < 0.05). Peer support predicted healthier eating behaviours among boys. Dietary resilience appeared to be influenced by both personal health values and the home food environment.	4/8 (moderate quality). Not met or unclear: standard criteria for outcome definition (4), strategies to address confounding (6), outcome measurement (7), statistical analysis (8).
del Mar Bibiloni et al. [36], Spain (Balearic Islands)	CS; *n* = 1231; 12–17 years; random sample.	Parental occupation‐based socio‐economic status and parental education.	Diet: 145‐item FFQ and two 24‐h dietary recalls. PA: leisure‐time SB (screen time).	Among girls, higher parental socio‐economic status was associated with the ‘Mediterranean’ pattern in multivariable models; associations of lower parental SES with the ‘Western’ pattern were significant only in crude analyses (girls, low vs. high SES: crude OR = 4.50; 95% CI, 1.72–11.75; adjusted OR = 3.75; 95% CI, 0.93–15.17). Boys with < 2 h/day of screen time were less likely to follow the ‘Western’ pattern (adjusted OR = 0.11; 95% CI, 0.03–0.47).	3/8 (low quality). Not met or unclear: inclusion criteria (1), SEP measurement (3), standard criteria for outcome definition (4), identification of confounders (5), statistical analysis (8).
Morley et al. [37], Australia	CS; *n* = 12,188; 12–17 years.	Neighbourhood SEP (SEIFA Index of Relative Socio‐economic Advantage and Disadvantage; low, medium, high).	Diet: fruit and vegetable intake, sugary drinks, and fast‐food consumption. PA/SB: MVPA (≥ 60 min/day) and screen time (< 2 h/day).	Adjusted proportions: compared with low‐SEP neighbourhoods, students from high‐SEP neighbourhoods more often met vegetable recommendations (27.2% vs. 22.5%) and the ≤ 2 h/day screen‐time recommendation (24.6% vs. 18.3%), and less often consumed sugary drinks daily (9.8% vs. 16.4%) or fast food weekly (35.3% vs. 47.4%). Meeting MVPA recommendations did not differ by SEP (*p* = 0.355).	8/8 (high quality). All criteria met.
Iglesia et al. [38], Europe (HELENA Study)	CS; 10 European cities; *n* = 2253 (diet) and *n* = 977 (biomarkers); 12.5–17.5 years.	FAS, maternal and paternal education, and maternal and paternal occupation (all entered in the same model).	Diet: two 24‐h dietary recalls. Biomarkers: plasma folate, serum vitamin B12, and holotranscobalamin concentrations.	Lower folate intake was associated with lower maternal and paternal education in both sexes, and lower vitamin B12 intake with almost all SEP indicators except paternal occupation. Lower plasma folate and vitamin B12 concentrations were also associated with lower paternal occupational status and lower maternal education.	5/7 (high quality). Not met or unclear: SEP measurement (3), statistical analysis (8). Item 4 not applicable (continuous outcome).
Stephens et al. [39], Australia	Long.; 2‐year follow‐up; *n* = 521; 12–15 years; socioeconomically disadvantaged adolescents.	Low maternal education (≤ 10 years schooling).	Diet: fruit (≥ 1 time/day) and vegetable consumption (≥ 2 times/day) assessed using an FFQ.	Being served vegetables at dinner was associated with higher vegetable intake (OR = 2.54; 95% CI, 1.57–4.10). Higher fruit intake was associated with female sex, family meal rules, and rarely buying from school vending machines (OR = 2.85; 95% CI, 1.34–6.07).	6/9 (moderate quality). Not met or unclear: outcome measurement (7), completeness of follow‐up (9), strategies for incomplete follow‐up (10). Items 1–2 not applicable (single socioeconomic stratum).
Stephens et al. [40], Australia	Long.; 2‐year follow‐up; *n* = 529; 12–15 years; socioeconomically disadvantaged adolescents.	Low maternal education (≤ 10 years schooling).	Diet: high‐energy‐dense foods and beverages assessed using an FFQ.	Lower consumption of high‐energy‐dense foods was associated with lower fast‐food intake as a main meal and reduced availability of these foods at home (*p* < 0.05). Presence of vegetables at dinner appeared protective against frequent consumption of high‐energy foods.	6/9 (moderate quality). Not met or unclear: outcome measurement (7), completeness of follow‐up (9), strategies for incomplete follow‐up (10). Items 1–2 not applicable (single socioeconomic stratum).
Finger et al. [41], Germany	CS; KiGGS Survey; *n* = 6359; 11–17 years; nationally representative sample.	Parental education adjusted for income and occupation.	Diet: fruit and vegetable intake, energy‐dense food consumption, and HuSKY score.	Higher parental education was associated with greater fruit intake among boys (OR = 2.0; 95% CI, 1.5–2.7) and girls (OR = 1.5; 95% CI, 1.0–2.1). Higher parental education was also associated with better HuSKY scores among boys (OR = 1.6; 95% CI, 1.2–2.2) and girls (OR = 1.5; 95% CI, 1.1–1.9).	7/8 (high quality). Not met or unclear: standard criteria for outcome definition (4).
Pisa et al. [42], South Africa (Agincourt)	CS; *n* = 388; Black adolescents from a rural area; 11–15 years.	Household SES (wealth index tertiles) and maternal education, obtained from the AHDSS.	Diet: nutrient patterns identified through PCA of 25 nutrients derived from a quantitative FFQ. Outcome: BMI‐for‐age *z*‐score.	Adolescents in the lowest (vs. highest) household SES tertile scored lower on the animal‐driven nutrient pattern (*β* = −0.34; *p* = 0.01) and higher on the mixed‐diet pattern (*β* = 0.17; *p* = 0.04); maternal education was not associated with any pattern. The animal‐driven pattern was positively associated with BMI‐for‐age *z*‐scores (*β* = 0.13; 95% CI, 0.02–0.24).	4/7 (moderate quality). Not met or unclear: SEP measurement (3), outcome measurement (7), statistical analysis (8). Item 4 not applicable (continuous outcome).
Stephens et al. [43], Australia	Long.; 2‐year follow‐up; *n* = 1201; 12–15 years; 37 schools in Victoria.	Maternal education (low: ≤ Year 10 vs. mid/high).	Diet: dairy consumption frequency assessed using a 38‐item FFQ. Additional intrapersonal and social correlates evaluated.	Frequent dairy consumption (≥ 1.5 times/day) was more common among mid/high‐ than low‐SEP adolescents at follow‐up (60% vs. 51%; *p* = 0.002) but not at baseline (51% vs. 46%; *p* = 0.079). SEP moderated two predictors: infrequently eating dinner alone and never buying from school vending machines predicted frequent consumption only among mid/high‐SEP adolescents.	7/11 (moderate quality). Not met or unclear: strategies to address confounding (5), outcome measurement (7), completeness of follow‐up (9), strategies for incomplete follow‐up (10).
del Mar Bibiloni et al. [44], Spain (Balearic Islands)	CS; *n* = 1231; 12–17 years; 53.6% girls.	Parental education and parental occupation‐based socioeconomic level.	Diet: KIDMED Index (Mediterranean diet adherence). PA: PA level (inactive vs. active).	In multivariable models, low (vs. high) parental education was associated with poor adherence to the Mediterranean diet among girls (OR = 3.38; 95% CI, 1.04–10.94), but not among boys; parental occupation‐based SES was not associated. Inactive girls were more likely to present low adherence (OR = 2.76; 95% CI, 1.52–5.02), while boys eating ≤ 3 meals/day also showed higher odds of poor adherence (OR = 4.14; 95% CI, 2.21–7.75).	2/8 (low quality). Not met or unclear: inclusion criteria (1), description of participants and setting (2), SEP measurement (3), identification of confounders (5), outcome measurement (7), statistical analysis (8).
Yannakoulia et al. [67], Greece	CS; school‐based study in low‐SES areas (DIATROFI); *n* = 15,897 children (3–12 years) and adolescents (13–18 years); FAS–diet associations reported separately for adolescents.	FAS and maternal/paternal education.	Diet: KIDMED score. PA: weekly sports participation.	Higher family affluence was associated with greater daily consumption of fruits, vegetables, dairy products, and breakfast (*p* < 0.001). Among low‐FAS adolescents, sports participation increased KIDMED scores by 12.4%. Lower FAS and lower parental education were associated with lower KIDMED scores.	4/7 (moderate quality). Not met or unclear: inclusion criteria (1), outcome measurement (7), statistical analysis (8). Item 4 not applicable (continuous outcome).
Michels et al. [68], Europe (HELENA Study)	CS; *n* = 2081; 12.5–17.5 years; 10 European cities.	Parental education, parental occupation, and FAS.	Diet: diet quality assessed using two 24‐h dietary recalls. Additional psychosocial mediation analyses performed.	Higher SES was associated with better diet quality (DQI‐A). Psychosocial factors explained 23%–64% of the association between SES and diet quality. Availability of soft drinks at home was the main mediator, accounting for 17%–44% of the total effect.	5/7 (high quality). Not met or unclear: SEP measurement (3), statistical analysis (8). Item 4 not applicable (continuous outcome).
Wadolowska et al. [61], Poland	CS; *n* = 1549; 11–13 years.	FAS; nutrition knowledge (not an SEP indicator).	Diet: 60‐item FFQ. PA: self‐reported PA frequency. Dietary/lifestyle patterns classified as ‘Prudent–Active’ and ‘Fast‐food–Sedentary.’	Moderate (vs. low) family affluence was associated with lower adherence to the ‘Fast‐food–Sedentary’ pattern (adjusted OR = 0.60; 95% CI, 0.42–0.86). Higher nutrition knowledge was associated with greater adherence to the ‘Prudent–Active’ pattern (OR = 2.40; 95% CI, 1.80–3.21). The ‘Fast‐food–Sedentary’ pattern was associated with a twofold higher risk of central obesity (OR = 2.22).	5/8 (moderate quality). Not met or unclear: standard criteria for outcome definition (4), outcome measurement (7), statistical analysis (8).
Borges et al. [66], Europe and Brazil	CS; HELENA and POF surveys; *n* = 1921 (Europe) and *n* = 14,344 (Brazil); 12.5–17.5 years.	Europe: FAS and parental education and occupation; Brazil: total family income and maternal education.	Diet: dietary patterns identified through factor analysis (Western and traditional patterns).	In European adolescents, the Western dietary pattern was associated with lower maternal education and, among boys, with higher FAS, whereas the traditional pattern was inversely associated with high FAS. In Brazilian adolescents, the Western pattern was associated with higher maternal education and higher SES, whereas the traditional pattern was more common among adolescents whose mothers had university‐level education.	3/7 (low quality). Not met or unclear: SEP measurement (3), identification of confounders (5), strategies to address confounding (6), statistical analysis (8). Item 4 not applicable (continuous outcome).
Vilela et al. [63], Portugal	CS (IAN‐AF survey); *n* = 1153; 3–17 years; adolescents (10–17 years) analyzed separately.	Socio‐economic composite classification (latent class analysis of parental education and employment, household composition, and region) and maternal and paternal education (separate models).	Diet: two 24‐h dietary recalls assessing fruit and vegetable intake, meat/fish/egg consumption, and snack intake.	Higher SES was associated with greater fruit and vegetable consumption among adolescents (*β* = 52.4; 95% CI, 9.6–95.3). Higher parental education was also associated with greater salty snack consumption. Adolescents from higher socioeconomic backgrounds consumed more fruits, vegetables, and higher‐quality protein sources.	7/7 (high quality). All criteria met. Item 4 not applicable (continuous outcome).
Esquius et al. [60], Spain	CS (DESKcohort); *n* = 7319; 12–18 years; 54.1% girls.	Perceived SEP (MacArthur Scale of Subjective Social Status, tertiles); educational track.	Diet: breakfast consumption (daily vs. skipping breakfast).	Adolescents with the most disadvantaged (vs. most advantaged) perceived SEP were more likely to skip breakfast daily in adjusted models. Associations were observed among girls (PR = 1.30; 95% CI, 1.11–1.54) and boys (PR = 1.28; 95% CI, 1.04–1.59).	5/8 (moderate quality). Not met or unclear: inclusion criteria (1), standard criteria for outcome definition (4), outcome measurement (7).
Marshall et al. [65], United States (Texas)	CS (Texas SPAN); *n* = 9056; 8th‐ and 11th‐grade adolescents; mean age 14.8 years.	School‐level SES tertiles (percentage of economically disadvantaged students); region (border vs. non‐border; not an SEP indicator).	Diet: fruit and vegetable variety score (0–7) and SPAN Healthy Eating Index.	Compared with the lowest school‐level SES tertile, adolescents in the highest tertile more often consumed ‘other vegetables’ (*p* = 0.039) but less often whole fruit (*p* < 0.001) and fruit juice (*p* = 0.013). FV variety was associated with healthier eating (mSHEI), most strongly in the highest SES tertile (e.g., FV variety: *β* = 0.81; *p* < 0.001).	2/7 (low quality). Not met or unclear: inclusion criteria (1), SEP measurement (3), identification of confounders (5), strategies to address confounding (6), statistical analysis (8). Item 4 not applicable (continuous outcome).
Belaoufi et al. [72], Morocco	CS; *n* = 386; 14–18 years; 61.6% girls.	Paternal and maternal education, paternal and maternal socio‐professional class, and monthly household income.	Diet: KIDMED index. PA (IPAQ short French version), screen time, sleep, BMI, and WHtR were also assessed but not analyzed by SEP.	In bivariate analyses, higher paternal and maternal education, higher parental socio‐professional class, and higher household income were associated with better KIDMED adherence (*p* ≤ 0.01). In the multinomial model including education and socio‐professional class of both parents (income not included), secondary or university paternal education was associated with lower odds of poor adherence (OR = 0.45; 95% CI, 0.23–0.87 and OR = 0.28; 95% CI, 0.12–0.66); maternal education was not associated, and the estimate for paternal socio‐professional class was unstable (empty cell). More than half of the sample showed poor adherence.	5/8 (moderate quality). Not met or unclear: SEP measurement (3), outcome measurement (7), statistical analysis (8).
Chavez‐Ugalde et al. [64], United Kingdom	Repeated CS (NDNS); *n* = 2991; 11–18 years; nationally representative sample collected over 11 years.	Parental occupation (NS‐SEC, three levels); geographic region (not an SEP indicator).	Diet: 4‐day food diaries; UPF consumption according to the NOVA classification (%TEI and g/day).	Mean energy intake from UPF was 65.9%. Adolescents whose parents had routine/manual (vs. higher managerial) occupations consumed a higher %TEI from UPF (+4.6%; 95% CI, 3.2–6.1), as did those with parents in intermediate occupations (+2.0%; 95% CI, 0.4–3.5). UPF consumption declined between 2008/09 and 2018/19.	7/7 (high quality). All criteria met. Item 4 not applicable (continuous outcome).
Colillas‐Malet et al. [59], Spain	CS (DESKcohort); *n* = 7319; 12–18 years; 54.1% girls.	Parental education (highest level) and perceived SEP (MacArthur scale, tertiles); educational track.	Diet: S‐HEI (outcome). PA assessed as a covariate.	In multivariable models, having parents with primary (vs. university) education was associated with lower S‐HEI scores among boys (*β* = −2.1; 95% CI, −3.1 to −1.1) and girls (*β* = −3.3; 95% CI, −4.3 to −2.3); perceived SEP was associated with S‐HEI only in bivariate analyses. Lower diet quality was observed among boys (*β* = −2.9; 95% CI, −4.3 to −1.5) and girls (*β* = −1.9; 95% CI, −3.5 to −0.3) enrolled in vocational education tracks.	4/7 (moderate quality). Not met or unclear: SEP measurement (3), outcome measurement (7), statistical analysis (8). Item 4 not applicable (continuous outcome).
Paul et al. [62], India (West Bengal)	CS; *n* = 442; adolescent girls; 10–19 years.	Household wealth score (PCA of assets) and father's education; caste/tribe (not an SEP indicator).	Diet: daily green leafy vegetable consumption, eating three meals/day, and IFA supplementation. PA: engagement in exercise (self‐reported, binary).	In mutually adjusted logit models, higher father's education was associated with engaging in exercise (*β* = 0.38; *p* < 0.01), and both household wealth (*β* = 1.46) and father's education (*β* = 0.75) with eating three meals/day (both *p* < 0.01); household wealth was not associated with green leafy vegetable consumption.	3/8 (low quality). Not met or unclear: inclusion criteria (1), description of participants and setting (2), SEP measurement (3), standard criteria for outcome definition (4), outcome measurement (7).
Shi et al. [70], China	CS; school‐based; *n* = 824; 12–14 years; Jiangsu Province.	Household possessions score (11 items) and paternal education.	PA: vigorous PA, household chores, and active commuting.	PA levels were higher among girls from lower‐SES backgrounds, mainly due to household chores. Active commuting was less common among boys from higher‐SES families and among those with highly educated fathers. In mutually adjusted ordinal models, high SES was associated with lower PA scores among boys (OR = 0.44; 95% CI, 0.24–0.82), and marginally among girls (OR = 0.53; 95% CI, 0.27–1.05); vigorous PA did not differ by SES. Among girls, higher SES and paternal education were associated with less TV viewing.	2/8 (low quality). Not met or unclear: inclusion criteria (1), SEP measurement (3), standard criteria for outcome definition (4), identification of confounders (5), outcome measurement (7), statistical analysis (8).
Boone‐Heinonen et al. [71], United States	CS (Add Health Study); *n* = 17,294 adolescents (Wave I).	Neighbourhood socioeconomic environment factors (advantageous economic environment: median household income, poverty, education; disadvantageous social environment: crime).	PA: MVPA analyzed in relation to GIS‐based environmental characteristics.	Among boys, the most advantaged economic environment was associated with more MVPA (Exp(coeff) = 1.07; 95% CI, 1.01–1.14), but not after further adjustment for the social environment (1.06; 95% CI, 0.98–1.14). MVPA was negatively associated with more homogeneous neighbourhood landscapes (Exp(coeff) = 0.91; 95% CI, 0.86–0.96). Among girls, lower MVPA levels were associated with neighbourhoods with a higher concentration of fee‐based recreational facilities (Exp(coeff) = 0.92).	6/7 (high quality). Not met or unclear: outcome measurement (7). Item 4 not applicable (continuous outcome).
del Mar Bibiloni et al. [45], Spain (Balearic Islands)	CS; *n* = 1961; 12–17 years; 53.6% girls.	Parental education and parental occupation.	Diet: FFQ and Mediterranean diet adherence. PA: IPAQ‐A; ‘sedentary behaviour’ defined by the authors as < 300 min/week of MVPA (i.e., insufficient physical activity).	Insufficient MVPA was more prevalent among girls with low than high parental education (56.1% vs. 43.5%), but in multivariable models, neither parental education (girls: OR = 1.18; 95% CI, 0.76–1.82) nor parental profession level (girls: OR = 1.23; 95% CI, 0.74–2.06) was significantly associated in boys or girls.	5/8 (moderate quality). Not met or unclear: SEP measurement (3), standard criteria for outcome definition (4), statistical analysis (8).
De Cocker et al. [46], Europe (HELENA Study)	Multicenter CS; 10 European cities; *n* = 2780; 12.5–17.5 years.	FAS and maternal education.	PA: MVPA and psychosocial correlates related to PA.	Higher FAS was associated with more MVPA, but not with total PA; maternal education was not associated with MVPA or total PA. The FAS–MVPA association was mediated by stage of change, awareness, modelling, self‐efficacy, barriers, and environmental correlates (multiple‐mediator model: *t* = 6.2; *p* ≤ 0.001).	5/7 (high quality). Not met or unclear: description of participants and setting (2), SEP measurement (3). Item 4 not applicable (continuous outcome).
de Rezende et al. [47], Brazil	CS (PeNSE); *n* = 109,104 ninth‐grade students; nationally representative sample.	Maternal education and household vehicle ownership (material asset); school type (public vs. private).	PA domains: leisure‐time PA, active commuting, and participation in physical education classes. Diet: nutritional scores.	Only 29% of adolescents met PA recommendations (≥ 300 min/week). Leisure‐time PA was the strongest contributor to meeting recommendations (PR = 12.5; 95% CI, 11.17–13.97). In mutually adjusted Poisson models, adolescents whose mothers had completed higher (vs. incomplete elementary) education were more likely to meet PA recommendations (PR = 1.20; 95% CI, 1.14–1.26), and household vehicle ownership was associated with leisure‐time PA (PR = 1.04; 95% CI, 1.03–1.05); public‐school students were less likely to meet recommendations (PR = 0.86; 95% CI, 0.78–0.94).	6/8 (high quality). Not met or unclear: SEP measurement (3), statistical analysis (8).
Scholes and Mindell [48], England and the United States	CS (HSE and NHANES); *n* = 8331; 11–17 years.	Household income level.	PA: MVPA including sports, recreational activities, informal activities, and active travel.	Adolescents from higher‐income households were more likely to participate in formal sports and exercise activities in England, both among boys (+11%; 95% CI, 4%–17%) and girls (+13%; 95% CI, 6%–20%). In the United States, recreational activity was greater among high‐income adolescents (boys: +15 min/day; girls: +19 min/day). In contrast, low‐income adolescents spent more time in informal activities (English girls: +21 min/day) and active travel (English boys; US boys and girls), so that the direction of inequality varied by PA domain.	6/7 (high quality). Not met or unclear: outcome measurement (7). Item 4 not applicable (continuous outcome).
Ren et al. [69], China (Nanjing)	CS; *n* = 7097; 12–15 years; 50.1% girls.	Parental education and area of residence (urban vs. rural).	PA: participation in organized sports through clubs or teams.	Only 16.6% of adolescents participated in organized sports. Boys were more likely to participate than girls (OR = 1.34; 95% CI, 1.18–1.53). No significant association was observed between parental education and organized sports participation.	2/8 (low quality). Not met or unclear: inclusion criteria (1), SEP measurement (3), standard criteria for outcome definition (4), strategies to address confounding (6), outcome measurement (7), statistical analysis (8).
Mori et al. [49], Japan	Multilevel CS; *n* = 8102; mean age 13.1 years; 50.5% boys.	Neighbourhood SES indicators, including average annual income, area deprivation, and neighbourhood educational level.	PA: MVPA assessed using an adapted IPAQ for Japanese youth and participation in organized sports.	Neighbourhood‐level SES indicators (each in a separate model) were not associated with organized sports participation or MVPA. They only moderated the relative age effect: relatively younger boys in low‐income neighbourhoods and younger girls in more deprived neighbourhoods reported less MVPA.	7/7 (high quality). All criteria met. Item 4 not applicable (continuous outcome).
Woodforde et al. [50], Australia	CS (LSAC); *n* = 3201; 12–13 years.	Weekly parental income and area‐level SES (IRSAD quintiles); family structure and other sociodemographic characteristics.	PA: before‐school PA assessed using 24‐h time‐use diaries, including active commuting, location, and social context.	Adolescents accumulated a mean of 10.8 min/day of PA before school. Boys and adolescents from single‐parent households were more likely to engage in before‐school PA, particularly through active commuting with peers. Neither weekly parental income nor area‐level SES was associated with before‐school PA.	6/7 (high quality). Not met or unclear: statistical analysis (8). Item 4 not applicable (continuous outcome).
Grasaas et al. [51], Norway	CS; school‐based; *n* = 90,091; 16–19 years; nationally representative sample.	Ungdata composite SES index (0–3; perceived family economy, parental education, and material prosperity; derived from FAS II).	PA: participation in organized sports (athlete vs. dropout). Additional outcomes: school stress, fatigue, and sense of belonging.	Current sport participants had higher SES than dropouts (2.13 vs. 1.97; *p* < 0.01), and dropouts decreased across SES levels (lowest 80.2%, middle 73.7%, highest 63.2%). Tennis participation was more common among higher‐SES adolescents, whereas motorsport was more common among lower‐SES adolescents. Adolescents engaged in sports participation were less likely to report school stress (OR = 0.83; 95% CI, 0.81–0.86).	1/8 (low quality). Not met or unclear: inclusion criteria (1), description of participants and setting (2), standard criteria for outcome definition (4), identification of confounders (5), strategies to address confounding (6), outcome measurement (7), statistical analysis (8).
Haddad and Sarti [52], Brazil	Repeated CS (PeNSE 2009, 2012, and 2015); ninth‐grade students from Brazilian state capitals; *n* = 60,973 (2009), 61,145 (2012), and 50,442 (2015).	Composite socioeconomic stratum (low, middle, high; score based on household durable goods, household conditions, and maternal education); school type (not an SEP indicator).	Diet: consumption of ≥ 3 healthy and ≥ 3 unhealthy food groups/day. PA: ≥ 60 min/day of PA.	In logistic models (reference: high stratum in 2015), the high stratum was more likely to meet PA recommendations in 2009 (*β* = 0.31 vs. *β* = −0.20 for the low stratum), and the low and middle strata were less likely to do so in 2015 (*β* = −0.82 and *β* = −0.70); PA declined in all strata from 2009 to 2015. No consistent socioeconomic gradient was observed for healthy food consumption, whereas lower strata were less likely to consume ≥ 3 unhealthy food groups/day (e.g., 2015: low *β* = −1.27; middle *β* = −0.98). Sedentary behaviour was not analyzed by SEP.	5/8 (moderate quality). Not met or unclear: SEP measurement (3), standard criteria for outcome definition (4), statistical analysis (8).
Libuy et al. [53], United Kingdom	Long. (Millennium Cohort Study); *n* = 7220; follow‐up from 5 to 17 years (2006–2018).	Permanent family income (quintiles of equivalised weekly net income from 9 months to 5 years); home ownership (wealth proxy) used only in adiposity models.	Diet: daily breakfast consumption. PA: ≥ 5 days/week of PA (MVPA from age 14). Also BMI and body fat.	At age 17, 54.7% of adolescents in the highest vs. 31.1% in the lowest income quintile ate breakfast every day, with income gaps present throughout childhood and adolescence. Income differences in regular PA at ages 7 and 11 narrowed by age 14 and were absent at age 17. Sedentary behaviour was not assessed.	6/11 (moderate quality). Not met or unclear: identification of confounders (4), strategies to address confounding (5), outcome status at baseline (6), outcome measurement (7), completeness of follow‐up (9).
da Costa et al. [54], Brazil	CS; *n* = 522; 14–18 years; 54.3% girls.	SES score based on household goods (0–100; nationally standardized asset index); family structure (not an SEP indicator).	PA/SB/Sleep: 24‐h movement behaviours assessed using wrist‐worn accelerometers. Diet: processed and unprocessed food consumption.	Greater processed food consumption was associated with higher SB (*β* = 3.73; 95% CI, 0.03–7.38) and lower MVPA (*β* = −0.89; 95% CI, −1.68 to −0.10). Girls also presented lower MVPA levels than boys (*β* = −4.76; 95% CI, −7.48 to −1.96). SES was not associated with sleep, sedentary behaviour, light PA, or MVPA (e.g., MVPA: *β* = 0.02; 95% CI, −0.12 to 0.16).	8/8 (high quality). All criteria met.
da Costa et al. [55], Brazil	CS; *n* = 731; mean age 16.4 years; 51% girls.	SES score based on household goods (0–100); family structure (not an SEP indicator).	PA/SB/Sleep: self‐reported 24‐h movement behaviours. Diet: processed and unprocessed food consumption.	Higher SES was associated with more total PA (*β* = 0.30; 95% CI, 0.07–0.53) but not with sports, non‐sport PA, sedentary behaviour, or sleep. Greater processed food consumption was associated with increased SB and lower PA levels.	7/7 (high quality). All criteria met. Item 4 not applicable (continuous outcome).

*Note:* Results are presented as measures of association with corresponding 95% confidence intervals whenever available. Studies were grouped according to their primary outcome domains (dietary behaviours, physical activity/sports participation, and combined lifestyle‐related outcomes). Methodological quality was assessed using the Joanna Briggs Institute (JBI) Critical Appraisal Checklists appropriate for each study design, and JBI scores are presented as the number of fulfilled criteria over the total number of applicable criteria. Quality was classified as high (≥ 70% of applicable criteria met), moderate (50%–69%), or low (< 50%); numbers in parentheses identify the checklist items rated as not met or unclear (item‐level ratings in Additional File S2). Sociodemographic variables that are not conventional SEP indicators (e.g., urban/rural residence, school type, ethnicity, family structure, geographic region, nutrition knowledge, and educational track) are listed in the ‘SEP Indicators’ column as reported by the original studies but were not treated as SEP indicators in the synthesis.

Abbreviations: AHDSS, Agincourt Health and Demographic Surveillance System; BMI, body mass index; CI, confidence interval; CS, cross‐sectional study; DQI‐A, Diet Quality Index for Adolescents; DQI‐I, Diet Quality Index‐International; FAS, Family Affluence Scale; FFQ, Food Frequency Questionnaire; FV, fruit and vegetables; GIS, Geographic Information System; HELENA, Healthy Lifestyle in Europe by Nutrition in Adolescence; HSE, Health Survey for England; HuSKY, Healthy Nutrition Score for Kids and Youth; IAN‐AF, National Food, Nutrition and Physical Activity Survey; IFA, iron‐folic acid; IPAQ, International Physical Activity Questionnaire; IPAQ‐A, International Physical Activity Questionnaire for Adolescents; IRSAD, Index of Relative Socio‐economic Advantage and Disadvantage; KIDMED, Mediterranean Diet Quality Index for children and adolescents; KiGGS, German Health Interview and Examination Survey for Children and Adolescents; Long., longitudinal study; LSAC, Longitudinal Study of Australian Children; mSHEI, modified SPAN Healthy Eating Index; MVPA, moderate‐to‐vigorous physical activity; NDNS, National Diet and Nutrition Survey; NHANES, National Health and Nutrition Examination Survey; NOVA, food classification system based on food processing; NS‐SEC, National Statistics Socio‐economic Classification; OR, odds ratio; PA, physical activity; PCA, principal component analysis; PeNSE, National School Health Survey; POF, Brazilian Household Budget Survey; POR, prevalence odds ratio; PR, prevalence ratio; SB, sedentary behaviour; SEIFA, Socio‐Economic Indexes for Areas; SEP, socioeconomic position; SES, socioeconomic status; S‐HEI, Spanish Healthy Eating Index; SPAN, School Physical Activity and Nutrition Survey; TEI, total energy intake; UPF, ultra‐processed foods; WHtR, waist‐to‐height ratio.

Dietary behaviours were the most frequently investigated outcomes, followed by physical activity and sedentary behaviour. Considerable heterogeneity was observed in the indicators of SEP, behavioural outcomes, and assessment methods. The specific SEP indicators, instruments, and outcomes assessed in each study are detailed in Table 1.

### 3.3 Socioeconomic Inequalities and Dietary Behaviours

Dietary behaviours were examined in 26 studies [18, 33, 34, 35, 36, 37, 38, 39, 40, 41, 42, 43, 44, 52, 53, 59, 60, 61, 62, 63, 64, 65, 66, 67, 68, 72], representing the largest group of studies included in this review. Adolescents with a higher SEP generally exhibited healthier dietary behaviours than those with lower SEP. Higher SEP was associated with greater fruit and vegetable consumption, better overall diet quality, and stronger adherence to healthy dietary patterns, including the Mediterranean diet [18, 36, 37, 41, 44, 59, 63, 67, 72].

Lower SEP was associated with less favourable dietary behaviours, including lower fruit and vegetable intake and more frequent breakfast skipping [18, 53, 60]. Several studies also found that adolescents with lower SEP were more likely to consume energy‐dense or ultra‐processed foods [37, 61, 64]. These findings indicate a generally consistent socioeconomic gradient in dietary behaviours across diverse settings. An exception was a Brazilian national survey, in which adolescents from lower socioeconomic strata were less likely to consume unhealthy food groups [52].

When findings were examined by type of SEP indicator, parental education showed the most consistent associations with diet. Higher parental education was associated with better diet quality or adherence to the Mediterranean diet [41, 44, 59, 67, 72], with more frequent fruit, vegetable, or dairy consumption [18, 41, 43], and with higher folate and vitamin B12 intake [38], and these associations generally persisted in models that also included other SEP indicators [38, 41, 59, 72]. However, some associations were restricted to one sex or to the education of one parent [44, 72], maternal education was not associated with nutrient patterns in rural South Africa [42], in Tunisian and Brazilian adolescents higher maternal education was associated with ‘modern’ or Western dietary patterns [35, 66], and in Portugal higher parental education was also associated with greater consumption of salty snacks [63]. Findings for parental occupation were mixed: routine or manual parental occupations were associated with higher ultra‐processed food intake in the United Kingdom [64], and higher occupation‐based SEP with a Mediterranean dietary pattern among girls in Spain [36], but occupation was not associated with adherence to the Mediterranean diet after adjustment in another Spanish study [44], and the corresponding estimate was unstable in a Moroccan study [72]. Higher family affluence was associated with more frequent consumption of fruit, vegetables, dairy products, and breakfast and with better diet quality [67], and moderate (vs. low) affluence with lower adherence to a fast‐food and sedentary lifestyle pattern [61], although among European boys, higher affluence was associated with a Western dietary pattern [66]. Household income or wealth was positively associated with daily breakfast consumption [53], regular meals [62], and an animal‐source nutrient pattern [42], but also with a ‘modern’ dietary pattern in Tunisia [35], and family income was not associated with dietary fat intake in a small study from the United States [33]. Among studies using composite or perceived measures, lower perceived SEP was associated with breakfast skipping [60], and a higher composite SEP with greater fruit and vegetable consumption [63], whereas perceived SEP was not associated with diet quality after adjustment for parental education [59], and lower socioeconomic strata consumed fewer unhealthy food groups in Brazil [52]. Finally, adolescents from higher‐SEP neighbourhoods more often met vegetable recommendations and less often consumed sugary drinks and fast food [37], whereas associations between school‐level SEP and fruit and vegetable consumption were mixed [65].

### 3.4 Socioeconomic Inequalities and Physical Activity

Physical activity was the main behavioural outcome in 14 studies [45, 46, 47, 48, 49, 50, 51, 52, 53, 54, 55, 69, 70, 71], and three further studies of dietary behaviours also reported physical activity by SEP [33, 37, 62]. Higher SEP was generally associated with greater participation in organized sports and higher levels of leisure‐time physical activity [48, 51]. Conversely, lower SEP was associated with a lower likelihood of meeting physical activity recommendations and with less moderate‐to‐vigorous physical activity [46, 47]. However, associations with total or moderate‐to‐vigorous physical activity were inconsistent. Positive associations were reported in five studies [46, 47, 52, 55, 62], whereas seven studies, including one using accelerometry, found no association between SEP and physical activity or sports participation [33, 37, 45, 49, 50, 54, 69]. In a UK birth cohort, income inequalities in regular physical activity observed at ages 7 and 11 narrowed by age 14 and were no longer present at age 17 [53]. Moreover, the direction of the gradient varied by domain: lower‐income adolescents engaged more in active travel or informal activities in England and the United States [48], and, in China, lower‐SEP adolescents were more physically active, mainly owing to housework and active commuting [70].

Few studies examined whether socioeconomic disparities in physical activity differed by sex, and no consistent pattern emerged. Overall, socioeconomic gradients were clearer for organized sports than for total physical activity, and their direction varied by physical activity domain and setting.

By type of SEP indicator, findings for physical activity were heterogeneous. Higher parental education was associated with meeting physical activity recommendations in Brazil [47] and with engaging in exercise in India [62], but it was not associated with physical activity or organized sports participation in three studies [45, 46, 69], and in China, higher paternal education was associated with less active commuting [70]. Higher household income was associated with participation in formal sports and exercise, whereas lower income was associated with more active travel and informal activity [48]; income was not associated with physical activity in two studies [33, 50], and income‐related differences were no longer present by late adolescence in one cohort [53]. For family affluence and household assets, higher affluence was associated with more moderate‐to‐vigorous but not total physical activity [46], household vehicle ownership with leisure‐time physical activity [47], and an asset‐based score with self‐reported total physical activity [55] but not with accelerometer‐measured activity in the same Brazilian study [54], while a higher household possessions score was associated with lower physical activity in China [70]. Higher composite SEP was associated with sport participation in Norway [51] and with meeting physical activity recommendations in Brazil [52]. Area‐level indicators were not associated with physical activity in three studies [37, 49, 50], and a positive association between neighbourhood economic advantage and moderate‐to‐vigorous physical activity among boys in the United States was attenuated after further adjustment [71].

### 3.5 Sedentary Behaviour

Socioeconomic differences in sedentary behaviour were examined in only four studies [37, 54, 55, 70]. Lower SEP was associated with more screen time in two of them: adolescents from low‐SEP neighbourhoods in Australia less often met the screen‐time recommendation [37], and, in China, higher household SEP and paternal education were associated with less television viewing among girls [70]. In contrast, two Brazilian studies, one using accelerometry, found no association between SEP and sedentary behaviour [54, 55].

Overall, the evidence on socioeconomic inequalities in sedentary behaviour was limited and inconsistent.

### 3.6 Quality Assessment

According to the JBI Critical Appraisal Checklists, 15 studies (39%) were classified as having high, 13 (34%) moderate, and 10 (26%) low methodological quality (Table 1; Additional File S2). Among the 33 cross‐sectional studies, most described participants and setting in detail (*n* = 28), identified confounding factors (*n* = 27), and used strategies to address them (*n* = 28). Fewer measured SEP (*n* = 15) or behavioural outcomes (*n* = 19) with instruments of demonstrated validity, defined categorical outcomes using standard criteria (5 of the 16 studies in which this item was applicable), or used statistical analyses that were clearly appropriate for the sampling design (*n* = 13); SEP was often reported by adolescents on behalf of their parents, and school‐based cluster samples were frequently analyzed without accounting for clustering. None of the five cohort studies was rated as high quality, mainly because follow‐up was incomplete or insufficiently described, and losses were rarely addressed analytically. The main methodological limitations included the predominance of cross‐sectional designs, reliance on self‐reported measures, and substantial heterogeneity in the assessment of SEP and behavioural outcomes. These limitations should be considered when interpreting the findings. Nevertheless, the overall pattern was similar when only the 15 high‐quality studies were considered: higher SEP was associated with healthier dietary behaviours in six of the seven such studies that examined diet [37, 38, 41, 63, 64, 68], the exception being a Tunisian study in which a ‘modern’ dietary pattern increased with household economic level [35], whereas findings for physical activity remained mixed [37, 46, 47, 48, 49, 50, 54, 55, 71].

## 4 Discussion

This systematic review synthesized evidence from 38 observational studies examining the association between SEP and adolescents' dietary behaviours and physical activity across diverse geographic settings. The included studies showed socioeconomic inequalities in adolescents' lifestyle behaviours. Adolescents with a higher SEP generally exhibited healthier dietary patterns and greater participation in organized physical activities. In contrast, those with lower SEP were more likely to present poorer diet quality and lower levels of leisure‐time physical activity, whereas associations with total physical activity and sedentary behaviour were inconsistent.

These results reinforce the importance of the social determinants of health in shaping behaviours established during adolescence, a critical developmental period with lasting implications for health across the life course [19, 20, 22]. Behaviours established during adolescence frequently persist into adulthood and contribute to the development of NCDs, thereby reinforcing socioeconomic inequalities in health over time.

One of the most consistent patterns identified across the included studies was the association between SEP and dietary behaviours. Adolescents with a higher SEP tended to consume more fruits and vegetables, achieve better overall diet quality, and show greater adherence to healthy dietary patterns, including the Mediterranean diet [18, 36, 37, 41, 44, 59, 63, 67, 72]. Conversely, adolescents with lower SEP were more likely to report lower fruit and vegetable intake, skip breakfast more frequently, and consume larger amounts of energy‐dense and ultra‐processed foods [18, 37, 53, 60, 64]. Diet quality was operationalized heterogeneously across studies, using a priori indices such as the KIDMED, the Healthy Eating Index, and dietary diversity scores, or a posteriori patterns (e.g., ‘Western’ and ‘Mediterranean’) derived from food frequency questionnaires or 24‐h recalls (Table 1); nonetheless, the socioeconomic gradient was generally observed with both types of measures.

These differences may reflect broader inequalities in food access, food affordability, nutrition knowledge, and neighbourhood food environments [14]. Families with greater socioeconomic resources generally have higher purchasing power, better access to fresh and nutritious foods, and greater health literacy, factors that may facilitate healthier dietary choices. In contrast, lower‐SEP families are more often exposed to food environments where healthy foods are less available or less affordable, whereas inexpensive ultra‐processed foods are more readily accessible. Such conditions may make healthy eating substantially more difficult.

Among the various indicators of SEP, parental education emerged as the indicator most consistently associated with healthier dietary behaviours. Higher parental educational attainment was associated with better diet quality and greater fruit and vegetable intake [18, 41, 44, 59, 72]. Previous research suggests that parental education may influence adolescents' behaviours through multiple pathways, including nutrition knowledge, health literacy, and the ability to establish healthier household routines and food environments [14, 22]. Consistent with these observations, supportive family environments may also promote dietary resilience, enabling some adolescents to maintain healthier eating behaviours despite limited socioeconomic resources [18].

Two non‐mutually exclusive explanations may account for this pattern. First, it may partly reflect the larger evidence base: parental education was the most frequently used indicator (20 of the 38 studies), which increases the likelihood of observing consistent associations. Education is also comparatively easy to measure, achieves high response rates, and is relevant regardless of employment status [23], so it may be measured with less error than income or occupation, particularly when SEP is reported by adolescents. Second, parental education may be uniquely relevant to diet, because it captures knowledge‐related assets that are largely acquired before parenthood and remain relatively stable over time [23], and that may shape food choices, nutrition literacy, and household food routines [14, 22]. The present data do not allow these explanations to be distinguished. Doing so would require more studies that examine several SEP indicators simultaneously in the same sample, so that their independent associations can be compared, as well as studies examining whether policies that expand parental education are followed by changes in adolescents' diets.

Although most studies associated lower SEP with less favourable dietary behaviours, one study suggested that some adolescents with lower SEP were able to maintain healthy eating patterns despite adverse socioeconomic circumstances, highlighting the concept of dietary resilience [18]. Protective influences within the family, school, and broader community may help explain this finding. At the same time, ongoing changes in food systems and the increasing availability of ultra‐processed foods may further widen dietary inequalities, particularly among lower‐SEP populations [10, 11, 12].

Despite the consistent socioeconomic gradient observed in dietary behaviours, some contextual differences emerged across countries at different stages of nutritional transition. In Tunisia and Brazil, higher SEP was associated with ‘modern’ or Western dietary patterns, possibly reflecting greater access to energy‐dense diets during earlier phases of economic development [21, 35, 66]. These observations suggest that the association between SEP and dietary behaviours is influenced by local social, economic, and nutritional contexts, reinforcing the need for public health strategies tailored to country‐specific realities.

Socioeconomic differences in physical activity were less consistent than those observed for diet. Adolescents with higher SEP were generally more likely to participate in organized sports and structured recreational activities [48, 51]. In contrast, adolescents with lower SEP were less likely to meet physical activity recommendations and reported less moderate‐to‐vigorous physical activity [46, 47]. Consistently, a meta‐analysis of studies from high‐income countries found that children and adolescents from higher‐SES households were more likely to participate in sport (OR: 1.87) and to meet physical activity guidelines (OR: 1.21), although no significant disparity was observed for leisure‐time physical activity [17]. In the present review, however, several studies found no association between SEP and physical activity or sports participation, including one using accelerometry [33, 37, 45, 49, 50, 54, 69], and lower‐SEP adolescents were sometimes more active through active commuting, informal activities, or housework [48, 70]. Financial constraints, limited access to recreational facilities, and unsafe neighbourhood environments may contribute to these disparities. However, a previous systematic review found no consistent evidence that the built environment mediates socioeconomic inequalities in physical activity among children and adolescents, and its moderating role was inconsistent across studies and regions [15]. Reducing these inequalities, therefore, requires strategies that extend beyond individual behaviour change and address broader social and environmental determinants of health.

Few of the included studies examined whether socioeconomic inequalities in physical activity differed by sex, and no consistent pattern emerged. Further research is warranted to clarify these associations and to better understand how socioeconomic and gender‐related factors jointly influence physical activity during adolescence. Similarly, SEP may interact with race and ethnicity. Evidence, mainly from the United States, indicates that the health returns of higher SEP tend to be smaller among racially and ethnically minoritized groups, a pattern described as ‘diminished returns’ [73, 74]. Few of the included studies examined such interactions, which therefore remain an important gap for future research.

Evidence on socioeconomic inequalities in sedentary behaviour was scarce and mixed: lower SEP was associated with more screen time in two studies [37, 70], whereas two studies, one using accelerometry, found no association [54, 55]. Although sedentary behaviour and physical inactivity are often examined together, they represent distinct behaviours with independent implications for health [28]. Physical inactivity denotes insufficient moderate‐to‐vigorous physical activity relative to guidelines, whereas sedentary behaviour refers to time spent sitting, reclining, or lying with low energy expenditure; an adolescent may therefore meet physical activity recommendations and still accumulate high levels of screen time, and each behaviour has been associated with health outcomes such as adiposity and cardiometabolic risk [9, 28]. The evidence synthesized in this review suggests that socioeconomic inequalities in physical activity and sedentary behaviour are less consistent than those in diet and depend on the behavioural domain and setting.

These findings are broadly consistent with previous systematic reviews. Lower SEP has been associated with poorer dietary behaviours among children and adolescents [75], whereas the association between SEP and physical activity was far from uniform, with 42% of studies reporting no association or an inverse one [76]. For sedentary behaviour, a meta‐analysis found that higher SES was associated with less sedentary behaviour in high‐income countries but with more non‐television screen time in low‐ and middle‐income countries [77], which may help explain the mixed findings observed in this review. However, the present review extends this evidence by incorporating studies conducted across a broader range of geographic and socioeconomic contexts and by including more recent publications. In addition, the included studies assessed SEP using diverse indicators, such as parental education, household income, family affluence, and neighbourhood characteristics. This broader evidence base provides a more comprehensive understanding of how socioeconomic inequalities are associated with adolescents' lifestyle behaviours while highlighting important contextual differences across populations.

The evidence across countries suggests that socioeconomic inequalities in adolescents' dietary behaviours are common, although the magnitude and expression vary according to local social, economic, and cultural contexts. For example, in high‐income settings, lower SEP was generally associated with lower fruit and vegetable intake and greater consumption of energy‐dense foods, whereas in some middle‐income countries at earlier stages of the nutrition transition, higher SEP was also linked to more ‘Western’ or ‘modern’ dietary patterns [21, 35, 66]. Differences in magnitude may also partly reflect measurement: adolescent‐reported indicators such as the FAS and parent‐reported education or income capture different dimensions of SEP and are subject to different reporting errors, and studies comparing extreme categories (e.g., highest vs. lowest tertile or quintile) tend to report larger differences than those using continuous scores or binary contrasts. This variability in exposure definitions, categorizations, and effect measures precluded direct comparison of the magnitude of associations across studies. These findings highlight the importance of developing evidence‐based public health strategies that remain responsive to the specific needs and realities of different populations.

The findings also have important implications for public health policy. Because socioeconomic inequalities in adolescent health behaviours are likely shaped by structural and environmental conditions rather than individual choices alone, effective interventions should extend beyond health education. Policies that improve access to affordable healthy foods, strengthen neighbourhood food environments, expand opportunities for organized sports, and provide safe spaces for physical activity may help reduce socioeconomic inequalities during adolescence. Likewise, actions that regulate the marketing and availability of ultra‐processed foods, together with school‐based health promotion initiatives, may contribute to healthier behaviours and more equitable health outcomes [12, 19, 22].

This review has several strengths. It synthesized evidence from a broad range of countries and considered multiple indicators of SEP, providing a comprehensive overview of how socioeconomic inequalities relate to adolescents' dietary behaviours and physical activity across diverse settings. In addition, methodological quality was assessed systematically using the JBI critical appraisal checklists, and item‐level ratings are reported transparently (Additional File S2).

Several limitations should also be acknowledged. Most included studies (31 of 38) employed cross‐sectional designs, limiting conclusions regarding causal relationships. Only 15 of the 38 studies were rated as having high methodological quality, mainly because SEP and behaviours were often self‐reported with instruments of unknown validity and because analyses frequently did not account for clustered sampling. Moreover, several included articles analyzed the same underlying samples (the HELENA study [38, 46, 66, 68], the Balearic Islands survey [36, 44, 45], the Youth Eating Patterns study [18, 39, 40, 43], the DESKcohort project [59, 60], one Brazilian school‐based study [54, 55], and the Brazilian National School Health Survey [47, 52]), so the number of independent samples is smaller than the number of included articles. Considerable heterogeneity was observed in the assessment of SEP and behavioural outcomes, reducing comparability across studies. Furthermore, many studies relied on self‐reported measures of diet and physical activity, which may be subject to recall and social desirability bias. In addition, few studies examined whether associations between SEP and adolescents' behaviours differed by race or ethnicity, despite evidence that these dimensions interact [73], and several studies reported sociodemographic variables that are not conventional SEP indicators (e.g., school type or urban/rural residence), which were not considered in our synthesis. Restricting inclusion to studies published in English or Portuguese may have led to the omission of relevant evidence, and because the search strategy combined dietary and physical activity terms with the AND operator, studies addressing only one of these behaviours may have been missed. Finally, the PubMed search exceeded the database export limit of 10,000 records, so records beyond this limit were not screened, and relevant studies may have been missed. Nevertheless, the consistency of the dietary findings across diverse populations and contexts supports the conclusion that SEP is associated with adolescents' dietary behaviours.

Future research should prioritize longitudinal studies to better understand the pathways through which SEP influences adolescents' dietary behaviours, physical activity, and sedentary behaviour over time. Greater standardization in the measurement of socioeconomic indicators and behavioural outcomes would also improve comparability across studies and strengthen future evidence syntheses. Such standardization should apply not only to the operationalization of SEP but also to the measurement of diet, physical activity, and sedentary behaviour. Most included studies relied on self‐reported data collected at a single time point, and findings sometimes differed by measurement method, as illustrated by an association between SEP and self‐reported but not accelerometer‐measured physical activity in the same Brazilian study [54, 55]. Future studies should therefore combine validated questionnaires with objective measures of physical activity and sedentary behaviour, use multiple 24‐h recalls, food records, or daily diaries to assess diet, and collect repeated measurements over time. Additional research from low‐ and middle‐income countries remains particularly important, especially in settings undergoing rapid nutritional and social transitions. Beyond the stage of economic development, social policies may also shape the magnitude of socioeconomic inequalities in adolescents' behaviours. Universal school meals, family income support, and publicly funded sports and recreational facilities, which are more common in comprehensive welfare states, could reduce the extent to which adolescents' diet and physical activity depend on household resources. The included studies were too few and too heterogeneous to compare countries by welfare regime, and future research should examine whether socioeconomic gradients in adolescent health behaviours are attenuated in settings with more extensive social protection. Such evidence may inform the development of more effective and equitable public health strategies to promote healthy lifestyles and reduce socioeconomic inequalities across the life course.

## 5 Conclusion

This systematic review indicates that SEP is consistently associated with adolescents' dietary behaviours across diverse geographic settings, whereas associations with physical activity and sedentary behaviour are less consistent and vary by domain and setting. The socioeconomic gradient was most evident for diet, with adolescents with lower SEP showing less favourable dietary behaviours.

These inequalities are likely shaped by interacting factors operating at the individual, family, community, and structural levels. Parental education was the indicator most consistently associated with adolescents' dietary behaviours, whereas evidence for the other indicators, including area‐level measures, was more heterogeneous.

Reducing these inequalities will require public health strategies that extend beyond individual behaviour change and address the broader social and environmental conditions influencing healthy lifestyles. Policies that improve access to nutritious foods, strengthen neighbourhood food environments, expand opportunities for safe physical activity, and reduce socioeconomic barriers to healthy living may contribute to healthier behaviours during adolescence and to reducing health inequalities across the life course.

Although additional longitudinal studies are needed to clarify the pathways underlying these associations, the evidence across countries highlights socioeconomic inequalities in adolescents' dietary behaviours as a persistent global public health challenge. Addressing these disparities is essential to promote healthier developmental trajectories and reduce the future burden of NCDs.

## Author Contributions

**Mariana R. Haddad:** conceptualization, methodology, investigation, data curation, formal analysis, validation, writing – original draft, writing – review and editing. **Flávia M. Sarti:** conceptualization, methodology, investigation, data curation, formal analysis, validation, supervision, writing – original draft, writing – review and editing. Both authors read and approved the final manuscript.

## Funding

The authors have nothing to report.

## Ethics Statement

Ethical approval was not required because this study was based exclusively on previously published studies and did not involve human participants.

## Conflicts of Interest

The authors declare no conflicts of interest.

## Declaration on the Use of Generative AI in Scientific Writing

During the preparation of this manuscript, the authors used ChatGPT (OpenAI) to assist with language editing and improvement of writing clarity. The authors reviewed and edited the output as necessary and take full responsibility for the content of this publication.

## Supporting information


Supporting File

## Data Availability

This study is based entirely on data reported in previously published studies. All sources are cited in the reference list, and no new datasets were generated.
