# Supplementary material for: Socioeconomic Inequalities in Dietary and Physical Activity Behaviours Among Adolescents: A Systematic Review
Source: J Hum Nutr Diet. 2026 Nov 2;39(6):e70374. doi: 10.1111/jhn.70374 (PMC13728465; doi:10.1111/jhn.70374)
Supplement: Supplementary file 1 [file JHN-39-0-s001.docx]

**Additional file 1:** Database search strategies.

Detailed search strategies used in PubMed/MEDLINE, Scopus, Embase, and BIREME (LILACS). Searches were conducted in February 2026 and combined controlled vocabulary terms (MeSH) and free-text keywords related to adolescents, socioeconomic status (SES), dietary behaviors, and physical activity. Search strategies were adapted to the indexing terms and search features of each database.

**PubMed / MEDLINE**

Date of search: February 15, 2026.

("Adolescent"[MeSH] OR adolescent* OR teenager* OR youth OR "young people" OR "young person")
AND
("Socioeconomic Factors"[MeSH] OR "socioeconomic status" OR SES OR income OR "household income" OR education OR "parental education" OR "family income" OR "social class" OR "socioeconomic position")
AND
("Diet"[MeSH] OR "Feeding Behavior"[MeSH] OR diet* OR "dietary habits" OR "dietary pattern*" OR "food consumption" OR "eating behavior" OR nutrition OR "food intake")
AND
("Motor Activity"[MeSH] OR "Exercise"[MeSH] OR "physical activity" OR exercise OR "physical exercise" OR "sedentary behavior" OR "sedentary lifestyle" OR inactivity)

## **Scopus**

Date of search: February 15, 2026.

TITLE-ABS-KEY(adolescent* OR teenager* OR youth OR "young people" OR "young person")
AND
TITLE-ABS-KEY("socioeconomic status" OR SES OR income OR "household income" OR education OR "parental education" OR "family income" OR "social class" OR "socioeconomic position")
AND
TITLE-ABS-KEY(diet* OR "dietary habits" OR "dietary pattern*" OR "food consumption" OR "eating behavior" OR nutrition OR "food intake")
AND
TITLE-ABS-KEY("physical activity" OR exercise OR "physical exercise" OR "sedentary behavior" OR "sedentary lifestyle" OR inactivity)

**Embase**

Date of search: February 15, 2026.

('adolescent'/exp OR adolescent* OR teenager* OR youth OR 'young people')

AND

('socioeconomic factor'/exp OR 'socioeconomic status' OR income OR education OR 'social class' OR 'socioeconomic position')

AND

('diet'/exp OR 'feeding behavior'/exp OR diet* OR 'dietary habits' OR 'dietary pattern*' OR 'food consumption' OR nutrition OR 'food intake')

AND

('physical activity'/exp OR exercise/exp OR 'physical activity' OR exercise OR 'sedentary behavior' OR 'sedentary lifestyle' OR inactivity)

**BIREME (LILACS)**

Date of search: February 15, 2026.

(adolescent* OR teenager* OR youth)

AND

("socioeconomic status" OR income OR education OR "social class")

AND

(diet* OR "dietary habits" OR "food consumption" OR "dietary patterns")

AND

("physical activity" OR exercise OR "sedentary behavior")
